# Trauma-analogue symptom variability predicted by inhibitory control and peritraumatic heart rate

**DOI:** 10.1038/s41598-025-99564-x

**Published:** 2025-04-30

**Authors:** Linn Petersdotter, Lindsey Miller, Mikael Johansson, Åsa Hammar

**Affiliations:** 1 Department of Psychology, Faculty of Social Sciences, Lund University, Lund, Sweden; 2 Department of Clinical Sciences Lund, Psychiatry, Faculty of Medicine, Lund University, Lund, Sweden; 3 Department of Psychiatry, Skåne University Hospital, Lund, Sweden

**Keywords:** Trauma film paradigm, Cognitive control, Heart rate, Intrusive memories, IES, Psychology, Emotion, Trauma, Cognitive control, Stress and resilience

## Abstract

The reasons why some individuals who experience trauma develop post-traumatic stress disorder (PTSD) while others do not remain poorly understood, highlighting the complex interplay of encoding-related and intrapersonal factors. This study aimed to examine factors predicting variability in trauma-related symptom development. Using a trauma film paradigm in a healthy sample (N = 32), we investigated how inhibitory control and peritraumatic responses relate to the development of intrusive memories and self-assessed event impact. Peritraumatic heart rate was associated with more frequent, vivid, and distressing memory intrusions during the week following trauma-analogue exposure. It also predicted hyperarousal and avoidance symptoms, with the latter further linked to lower inhibitory control. In a cognitive-interference task conducted approximately one day after trauma-analogue exposure, negative trauma reminders increased response latencies. This reduced interference control was predicted by both lower inhibitory control and higher peritraumatic heart rate, and it was especially pronounced in individuals who reported a heightened overall event impact. In conclusion, inhibitory control and peritraumatic heart rate emerged as predictors of subsequent reminder interference, intrusions, and self-assessed event impact. These findings provide insights into physiological and behavioural mechanisms underlying variability in the development of trauma-analogue symptoms and related cognitive interference when exposed to trauma reminders in a healthy sample without a trauma history.

## Introduction

Everyone experiences stressful life events, with over 70% of the population exposed to at least one traumatic incident^1^. While stressful events can encompass a range of experiences, trauma represents a more extreme subcategory defined by one of the leading diagnostic manuals for mental disorders as “[…] exposure to actual or threatened death, serious injury, or sexual violence […]”^2^. However, only a minority of approximately 6% will develop post-traumatic stress disorder (PTSD)^3^. The prevalence of clinically affected individuals who have experienced different types of trauma varies widely^1,4^. For instance, victims of sexual assault have a 50% likelihood of developing PTSD^5^, while following natural disasters, the rate of diagnosis can range from as low as 5% to as high as 60% of affected individuals^6^. The high diversity in responses to trauma implies significant individual differences in how people cope with a traumatic event^7^. Despite several empirically supported treatments^8^, only a subset of patients benefits from existing therapeutic interventions, while treatment resistance and drop-out rates remain high^7^. It is crucial to gain a deeper understanding of the underlying factors and mechanisms that contribute to an individual’s vulnerability to traumatic experiences, as such insights can help develop, duplicate and target treatment more efficiently^9^.

The primary objective of this study is to examine factors that predict individuals' responses to a distressing event (i.e., the exposure to trauma analogues). By doing so, we aim to understand why some individuals are more impacted by trauma than others. Further, this study seeks to explore how subjective (such as self-assessments of peritraumatic emotions and subsequent intrusions) and objective measures (such as peritraumatic heart rate and reminder interference control) capture the impact of trauma-analogue experiences in a non-clinical sample.

### PTSD: a memory disorder

While PTSD can manifest through a range of symptoms, it is primarily understood as a memory disorder^10^. The hallmark symptom of PTSD is intrusive memories—the relentless recurrence of unwanted and often vivid reexperiences of aspects of the trauma. Although most individuals experience some degree of intrusive memories after trauma, these tend to diminish over a few weeks^11,12^. However, it remains unclear why intrusions persist for some individuals, increasing their susceptibility to developing PTSD^13^. A recent review proposed that memory intrusions may play a key role in triggering other post-traumatic stress symptoms (PTSS), such as avoidance, arousal and reactivity, and negative alteration in cognitions and mood^11^.

Trauma-related intrusions, in contrast to non-traumatic and voluntarily recalled trauma memories, are marked by a sense of 'nowness,' heightened distress, and lack of contextual details—features associated with increased PTSD severity^14^. One explanation is that the excessive arousal evoked during trauma alters encoding and results in a poorly integrated sensory representation of the event^15,16^ that is readily reactivated by a wide range of seemingly unrelated or benign reminders (overgeneralisation)^10,17^. Given their importance in understanding the progression of the disorder, much of the research in the field uses the quality and quantity of memory intrusions as indicators of event impact^18^.

### The cost of trauma-related intrusions

The spontaneous re-experiencing of trauma during memory intrusions may also impair cognitive functioning. A recent systematic review and meta-analysis reported that when exposed to trauma-relevant reminders compared to neutral stimuli, PTSD patients displayed stronger interference effects on an emotional Stroop task^19^ with interference referring to the disruption of efficient task performance when confronted with a salient, yet task-irrelevant stimulus (e.g., a trauma-related image). The emotional Stroop paradigm is used to assess interference resolution through trials varying in cognitive control demands and emotional valence. While a large body of research has demonstrated a general processing cost for negative stimuli, several studies have reported an attention-mediated shortening of response times^20,21^. Notably, although greater interference was found in PTSD patients for trauma-related stimuli, this effect was not observed for negative stimuli in general, suggesting a modulation of cognitive control specifically in response to trauma reminders^19^. Recent research has further shown that combat veterans diagnosed with PTSD demonstrated extended response times to trauma-related cues compared to other negative or neutral stimuli, accompanied by elevated neural activity indicative of heightened arousal and attentional bias^22^.

### The study of susceptibility factors

In attempts to identify underlying susceptibility factors to trauma impact, researchers face the challenge of conducting post-trauma investigations with vulnerable individuals or recreating adverse events in controlled settings using trauma analogues. A widely used approach is the trauma film paradigm^18^, which exposes carefully selected healthy participants to a trauma-analogue experience while keeping stress levels subclinical.

### Does inhibitory control predict the impact of trauma?

Inhibitory control may play a crucial role in the development of trauma-related symptoms due to its importance in suppressing unwanted thoughts^23,24^. As a fundamental aspect of executive function, inhibitory control enables individuals to align thoughts and behaviours with internal objectives^25^. It is traditionally divided into response and cognitive inhibition^26^: The cognitive domain refers to an individual's capacity to regulate thoughts and emotions, such as suppressing a distressing memory from the past, whereas response control is associated with interrupting automatic motor actions, like refraining from reaching to catch a falling knife.

The notion that inhibitory control is functionally linked to the development of PTSD through its role in suppressing distressing thoughts is corroborated by recent research^23,27,28^. Individuals who survived a terror attack but did not develop PTSD exhibited superior inhibitory control compared to those with PTSD, even outperforming a closely matched non-trauma-exposed control group^27^. These findings suggest that inhibitory control can act as a protective factor in trauma exposure. Because it is difficult to determine whether inhibitory control is a preceding protective factor or a consequence of PTSD symptom pressure, experimental trauma paradigms can offer valuable insights by assessing participants’ baseline inhibitory control. To assess inhibitory control in the present study, we employed the Colour-Word Stroop task as a baseline measure. The Stroop task is widely used in psychological research to assess cognitive inhibition, as it requires individuals to suppress semantic information during automatic word reading in favour of naming ink colours^29^. This task provides a well-established measure of an individual's ability to override prepotent responses, a key aspect of inhibitory control, which has been linked to PTSD symptomatology in previous research^27,30^.

### Peritraumatic responsivity predicts PTSS

Supported by prior research and psychopathological models, peritraumatic responses (i.e., psychological and physiological reactions occurring during or directly after a traumatic event) can serve as informative indicators of the potential development of PTSS^31^. Research has found elevated heart rates in trauma-exposed individuals on their way to the hospital^32,33^ and in PTSD patients when recalling the trauma or when presented with reminders^34,35^. However, heart rate measures after the trauma or during recall may not fully capture the physiological reactivity experienced during the traumatic event itself. Studies utilising the trauma film paradigm have demonstrated increased heart rates in participants when exposed to trauma-analogue film clips compared to neutral scenes^36,37^. Additionally, heightened heart rate along with feelings of peritraumatic disgust has been shown to predict later intrusive memories in one study^37^, while another study found that only peritraumatic distress, not heart rate, predicted subsequent intrusions^36^.

Additional lines of research suggest that a decrease in heart rate during trauma reactivation^38^, temporally close to the traumatic incident^39,40^, or during trauma-analogue film viewing predicts PTSS, such as the frequency of intrusive memories^41^. PTSD patients who exhibit decreased heart rate during trauma recall tend to show less improvement from therapy over time, potentially indicating lowered emotional access to and, consequently, impaired processing of the trauma memory^38^.

While an increase in heart rate in response to negative, potentially threatening stimuli can be viewed as an adaptive physiological response, preparing the body for fight or flight^42,43^, a decrease in heart rate in the face of immediate threats or during the recollection of emotionally distressing memories may indicate a maladaptive reaction, implying a lack of emotional engagement and higher risk for dissociation^44,45^. Dissociative symptoms, such as derealization, depersonalization and emotional numbing, were found to predict PTSS and the severity of chronic PTSD^46^. The empirical evidence for the influence of these physiological reactions on predicting trauma-analogue symptoms such as intrusions remains, however, scarce^36^. In the present study, we combined objective measures of perifilm heart rate with subjective ratings of peritraumatic emotions.

### What underlies susceptibility to developing trauma-analogue symptoms?

We sought to understand how potential vulnerability-related factors may predict an individual’s response to trauma-analogue experiences. To ensure experimental control while exploring the expected variability in trauma-analogue symptom development, we selected a non-clinical sample for participation in a trauma-film study. Drawing from previous research and theory, we hypothesised that individuals’ inhibitory control and peritraumatic response (i.e., heart rate during trauma-analogue exposure and experienced emotions assessed immediately after film viewing) would predict trauma-analogue symptom development. We measured PTSD-analogue symptoms in three ways: (a) the frequency and quality (vividness and distress) of memory intrusions, assessed with a daily intrusion survey over six days following trauma-analogue exposure, (b) the self-assessed symptom pressure (Impact of Event scale-revised, IES)^47^ one week after trauma-analogue exposure, and (c) participants’ handling of trauma reminders in a cognitive-interference task 24–48 h post-exposure. For the cognitive-interference task, drawing from recent studies^19,21,22^, we expected trauma reminders to tax resources and consequently modulate response latencies.

## Methods

### Participants

Given the potentially distressing nature of the study, participants were carefully recruited through a digital pre-screening procedure. The pre-screening followed guidelines from previous research using the trauma film paradigm to ensure that unsuitable and potentially vulnerable individuals were excluded^48^ (a more detailed description of exclusion criteria and the selection process can be accessed in the supplementary materials). We collected data from 32 participants between the ages of 20 and 53 years (M = 28.6/SD = 6.9), with 23 individuals identifying as female. Our decision to include both male and female participants was driven by the aim of ensuring broader generalizability and counteracting the already reduced eligible participant pool due to adherence to ethical standards. Convenience sampling through word-of-mouth and social media platforms provided a group with varying cultural, professional, and social backgrounds. Participants were compensated with vouchers totalling a value of 300 Swedish kronor.

### Procedure

**Fig. 1:**
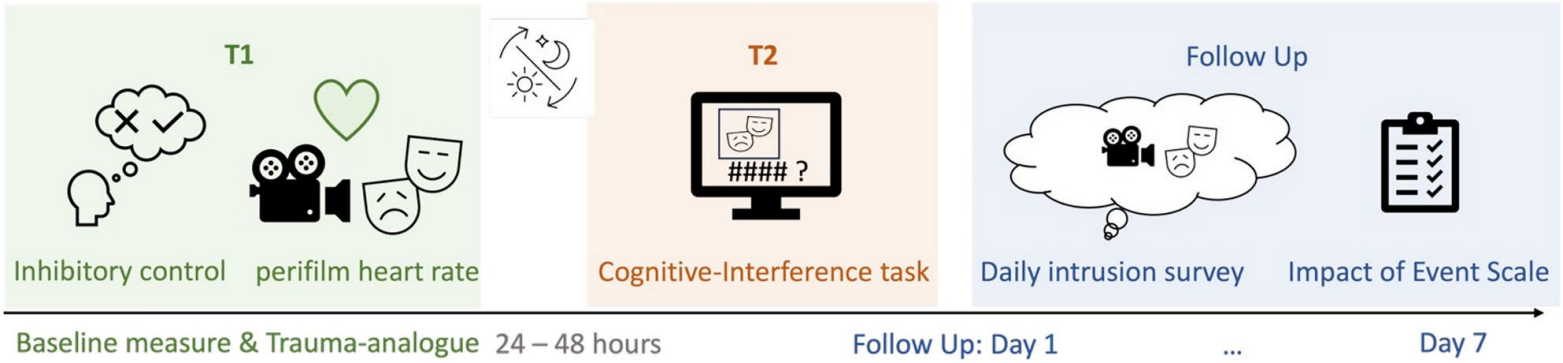
Visualization of trauma-analogue paradigm and follow up. *Note*. T1: After we measured participants’ baseline inhibition, they watched trauma-analogue and control scenes while their electrocardiogram was recorded. After each scene, participants rated the emotions felt during the exposure. T2: Participants came back to the lab after a delay for a cognitive-interference task in which reminders from the movie scenes or non-reminders were presented preceding trials which varied in cognitive control demands. From the first to the sixth day after trauma-analogue exposure participants reported potential intrusive memories. On day 7 participants filled out the Impact of Event scale.

The study consisted of two lab appointments with 24–48 h in between and a one-week follow-up of reporting potential intrusive memories. An overview of the study design is depicted in Fig. 1. T1 consisted of baseline measurements of inhibitory control and exposure to the film scenes. Upon arriving at the lab, participants were provided information about the study and the informed consent form. After giving written informed consent, participants were instructed to complete two tasks to assess inhibitory control: the Stroop Colour and Word Test^29^ and the Go/NoGo task^49^ in a counterbalanced order (see supplementary materials for a detailed description of respective tests). Participants were again verbally informed about the potentially distressing nature of the upcoming movie scenes, after which they were prepared for the electrocardiogram (ECG) recording with electrode leads to the participant’s wrists and neck, consistent with the research protocols adopted in the lab (see supplementary materials for a detailed description of psychophysiological measurements and laboratory equipment).

Before exposure to the movie scenes, participants were instructed to keep their visual focus on the screen and watch as if they were witnesses observing the events unfolding before them. Audio was played through headphones.

The two trauma-analogue scenes selected from the movie *Irréversible*^50^ portrayed a sexual assault and a murder, respectively, exclusively representing interpersonal trauma to maintain consistency in the nature of the trauma across participants. Additionally, interpersonal trauma has been shown to elicit stronger psychological impact compared to non-interpersonal trauma^51^. The movie has previously been utilised in various studies employing the trauma film paradigm^37,52–54^. The control scene selected from the same movie displayed a mundane conversation in a subway. Each of the scenes played for 9 min. The order of the trauma-analogue scenes was counterbalanced, with the control scene always shown in the middle (see supplementary materials for a full description of the viewing material and procedure).

After viewing each film scene, participants completed a short questionnaire rating the emotions they experienced on a Likert scale ranging from 0 (not at all) to 10 (felt strongly). The rated emotions were *amusement, anger, fear, surprise, disgust, boredom* and *discomfort*. The scale was developed based on prior research employing comparable methodologies and the peritraumatic emotional responses reported by individuals with trauma histories^55,56^. When the film-watching concluded, participants were instructed about the intrusion surveys commencing the following day.

**Fig. 2:**
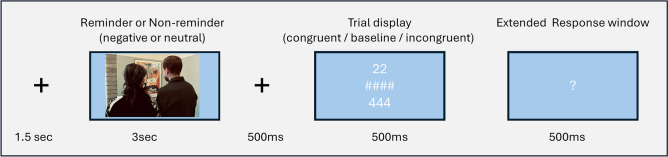
Example trial of the cognitive-interference task (T2). *Note*. The trial following the reminder or non-reminder would take either the form of a congruent, a baseline or an incongruent trial in a counter-balanced manner. For purpose of display examples of all conditions are shown. The task-irrelevant reminder / non-reminder, either negative or neutral, would be followed by a fixation cross. The subsequent task-relevant trial required the participant to quickly count the number of symbols presented. The trials varied in cognitive control demand, with congruent trials displaying digits matching the number of symbols, a baseline condition using hashtag symbols, and incongruent trials showing digits mismatched with the symbol count. The image shown is for illustrative purposes and was not part of the original stimulus material used in the study.

During T2, which was scheduled one to maximal two days after exposure, participants completed a cognitive-interference task which was inspired by the traditional Colour Word Stroop task^29^ and the Multi-Source Interference task (MSIT)^57^, with trials varying in cognitive control demands using digits and symbols as stimuli (see Fig. 2 for an example trial and the supplementary materials for the full description of conditions, trial-, block numbers and counterbalancing regime). Crucially, the trials included images that were irrelevant to the task but worked as reminders from the trauma-analogue scenes or previously unseen non-reminders. These additional images were included to capture potential additional interference elicited based on the mnemonic value (trauma-analogue reminder versus non-reminder) and the valence (neutral versus negative) while participants solved trials varying in cognitive control demands (congruent, baseline, incongruent).

During the one-week monitoring, participants were sent a digital daily intrusion survey capturing the frequency and quality (vividness and distress) of intrusions. Participants indicated the content of their intrusive memories by selecting from a predefined list of all the presented scenes or opting for “I do not remember”. Additionally, an open text box was provided for further comments and for describing potential reminders. These comments were not analysed further. On day 7 post-exposure, participants assessed their trauma-analogue symptoms with the IES, which consists of three subscales: *Avoidance, Intrusion*, and *Hyperarousal*^47^. Four weeks after T1, participants were individually contacted to ensure their well-being. No lasting effects were reported.

### Ethical considerations

The study was conducted in accordance with the Swedish Act concerning the Ethical Review of Research involving Humans (2003:460) and was approved by the Swedish Ethical Review Authority (reference number 2022-05425-01). All personal data collected throughout the study was pseudonymised and securely stored following the General Data Protection Regulations (GDPR).

### Data analysis

Behavioural data was analysed with R Studio^58^ and Jamovi^59^. Physiological data were visually inspected in AcqKnoweldge^60^ before potential inclusion and further pre-processed and analysed in Matlab^61^. In an a priori analysis (see also supplementary material), we tested reaction time (RT) differences for trial conditions varying in cognitive control demands (congruent, baseline, incongruent) during the cognitive-interference task with an analysis of variance (ANOVA). Trials with high control demands (i.e., the incongruent task condition) should capture PTSD symptomology of suffering interference with daily life tasks caused by potential trauma reminders. Further, our prediction that trauma-analogue reminders would cause additional interference visible in the modulation of RT latencies was followed up with another ANOVA (Primes [reminders versus non-reminders] x valence [negative versus neutral]). Based on these results we directly established RTs as a proxy for participants’ ability to handle cognitive control demands when reminded of the trauma-analogues instead of using a difference measure as a criterion for interference to avoid false positives^62,63^. To account for the skewed distribution of RT data, we log-transformed all RTs and analysed the median instead of the mean.

Our primary focus on factors that may underlie variability of trauma-analogue impact was investigated with hierarchical regression models with predictors entered in the temporal order of the experimental procedure, clustered in their respective categories (i.e., block 1: baseline measurement of inhibitory control, block 2: heart rate measured during trauma-analogue exposure, block 3: emotion ratings taken after the trauma-film viewing).

In a follow-up analysis, we explored whether self-assessed event impact related to the individual extent of reminder interference by splitting our sample based on the median of event impact into a more impacted group and a less impacted group. To account for differences in overall individual RT we compared the magnitude effect (ME = RT_incongruent_ − RT_baseline_)^21^ based on RTs for baseline and incongruent trials between the groups.

Potential autocorrelation of residuals, collinearity (Variance Inflation factor < 5 indicating low multicollinearities for all variables) and normality among predictor variables were tested, and residual plots and Quantile–Quantile-plots for standardised residuals were inspected visually. Since variables were measured on different scales, all predictor variables were z-standardized before submission to the regression models. For assumption testing and manipulation checks, ANOVAs and t-tests were used. To maintain a familywise-error rate of 0.05, alpha levels were adjusted using a Bonferroni correction where appropriate.

From participants’ ECG data, we calculated their mean heart rate for each movie scene, following previous research that employed similar paradigms^36,39,64,65^. For subsequent analyses, we included pre-selected 2-min sequences of continuous heart rate measurements instead of the entire 9-min recordings per scene. This approach was chosen to account for heart rate fluctuations at the beginning of each scene and to focus on isolating responses to the most traumatic moments. Specifically, these sequences featured a 2-min depiction of a violent rape in the assault scene and a brutal fight in the murder scene, culminating in the male character’s death from repeated blows with a fire extinguisher. For the control scene, we selected a 2-min segment from the middle of the scene, which showed the continuation of a trivial conversation among three actors in a metro. This was done to isolate changes in heart rate fluctuations associated with the trauma-analogue compared to the control scene. Two ECG datasets were disregarded due to technical failure, and two participants did not answer the final survey (IES) on day 7. Missing values were interpolated with the group mean to retain statistical sensitivity. One participant was excluded due to extremely noisy ECG data, resulting in a final sample of N = 31 (details about outlier exclusion, data preprocessing of the ECG, and behavioural data are available in the supplementary materials).

## Results

**Fig. 3:**
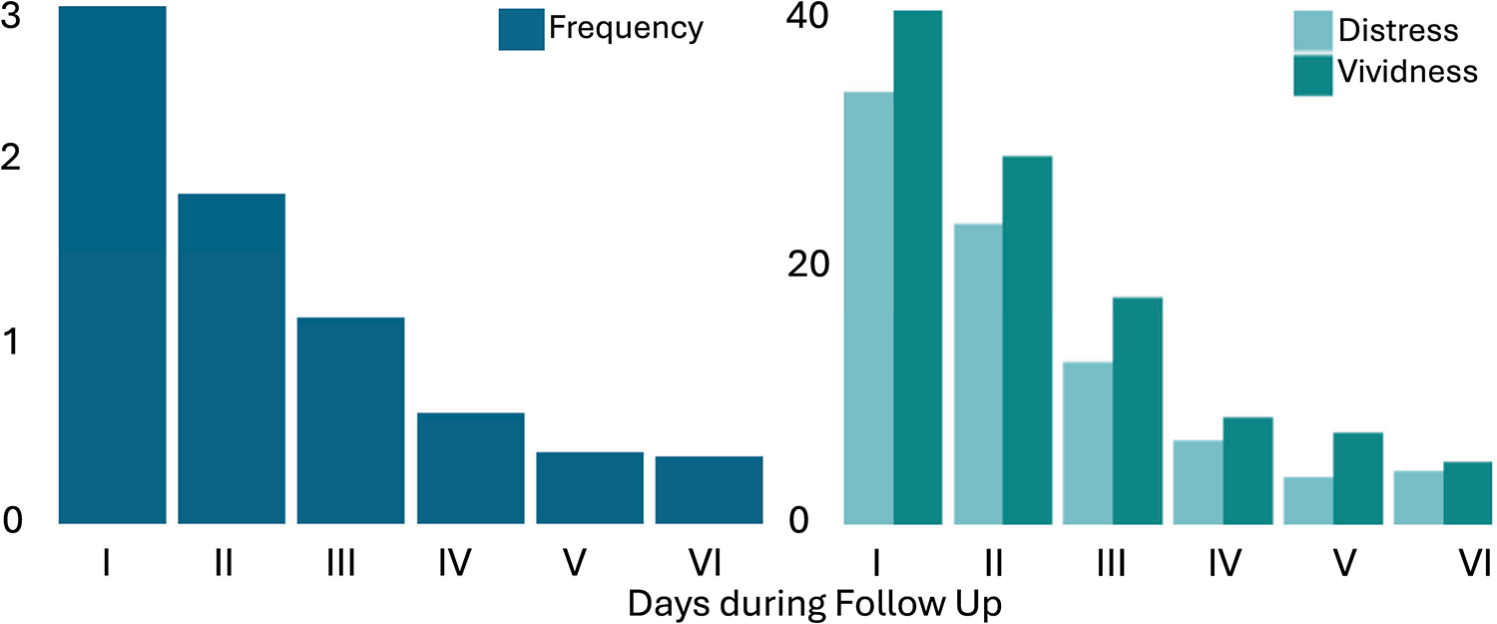
Mean Memory intrusion frequency and intrusion quality over six days following T1. *Note*. Ratings for intrusion frequency and quality are averaged over participants (frequency was measured with 0–4+; distress and vividness with a sliding scale from 0 to 100). Intrusions were assessed daily for six days, beginning 24 h after participants left the lab after the trauma-analogue exposure at T1.

Before proceeding to the primary analyses, we conducted a priori analyses to confirm key premises of the trauma-analogue paradigm (see supplementary material for details). These analyses confirmed that the trauma-analogue scenes were perceived as distressing and elicited different effects on heart rate, with the assault scene resulting in a significantly higher mean perifilm heart rate, while the murder scene showed a numerically lower mean heart rate compared to the control scene. The latter finding challenges the typical expectation that distressing stimuli lead to increased autonomic arousal. However, HRV analyses revealed no significant differences between the trauma-analogue scenes and the control condition (see supplementary material S8 and S9). To ensure our selected time windows captured trauma hotspots while controlling for effects specific to the start of each scene (such as apprehension), we compared heart rate between the initial and the later segments of each scene. Heart rate remained stable for the control and murder scenes but increased significantly from the beginning to the hotspot in the assault scene. These findings support our approach of analysing scene-specific hotspots rather than averaging across entire scenes or employing uniform time windows. The paradigm induced vivid and distressing intrusions, gradually declining over six days (see Fig. 3). In terms of content, 67% of all intrusions were related to the sexual assault scene, while 33% originated from the murder scene.

The two baseline measures of inhibitory control did not significantly correlate. As the Stroop score more closely reflects the cognitive component of inhibition and has previously been shown to distinguish symptom pressure in trauma-exposed individuals^19,27^, it was selected as the predictor for trauma-analogue event impact in subsequent analyses with higher scores reflecting lower inhibitory control.

We also examined the task conditions in the cognitive-interference task (T2) to confirm expected differences in processing speed. Overall accuracy was high (97%), with significantly slower performance (*p* < 0.001) for incongruent trials compared to both baseline and congruent trials, and slower RTs for baseline compared to congruent trials, indicating the intended variation in cognitive control demands. Lastly, analyses showed that participants with higher IES scores experienced significantly more intrusions than the less impacted group (*p* < 0.05). For readability, not all statistical coefficients are included here (see supplementary materials for a detailed overview, S1–S5). The descriptive statistics of variables included in the analyses are given in Table 1.

**Table 1:** Descriptive statistics of predictors and dependent variables.

Variable	Mean	Median	SD	Minimum	Maximum	Missing
Intrusion frequency	7	6	5.03	0	21	2
Intrusion distress	24.3	21	18.6	0	73	2
Intrusion vividness	28.5	26	22.1	0	81	2
IES score	13	10	11.5	0	44	2
IES avoidance	0.73	0.44	0.77	0	2.83	2
IES hyperarousal	0.31	0.22	0.36	0	1.20	2
IES intrusion	0.71	0.63	0.61	0	2.13	2
RT neu non-reminder	580	576	93.8	386	757	1
RT neg non-reminder	559	523	112	401	926	1
RT neu reminder	576	574	93.5	378	824	1
RT neg reminder	586	590	93.4	393	819	1
Inhibitory Control	17.2	16	7.46	7.5	37.5	0
Emotion murder	5.97	5.83	1.91	2.00	8.93	0
Emotion assault	8.38	8.40	1.32	5.20	10	0
HR murder	73.3	73.2	6.80	61.3	91.4	2
HR assault	78.8	77.7	10.2	62.3	111	2
HR control	74.5	74.6	6.79	62.0	88.8	2

HR stands for heartrate, RT for Reaction time in ms, neu for neutral, neg for negative, emotion murder and assault stand for the respective collapsed scores of the peritraumatic emotion rating scales. Intrusion distress and vividness was averaged over the first three days following trauma-analogue exposure. The column “missing” refers to missing datapoints which were interpolated with the group mean.

### Peritraumatic heart rate predicts intrusion frequency, vividness, and distress

We conducted three hierarchical multiple regressions to assess how well our predictor variables explained intrusion frequency, distress and vividness. For intrusion frequency, the initial model, which included only baseline inhibitory control, did not yield significant results. In the second step, adding peritraumatic heart rate from the two film scenes significantly improved the model (*p* < 0.005), though the effects differed in directions (*β*_AssaultScene_ = 0.730, *SE* = 1.050; *β*_MurderScene_ =  − 0.669, *SE* = 1.055). The final model, which also included peritraumatic emotion ratings for both scenes, explained 36% (*p* < 0.05) of the variance in intrusion frequency, though the emotion ratings did not significantly contribute (see supplementary Table S1).

Two separate regression analyses investigated the quality of memory intrusions, specifically self-reported vividness and distress experienced during trauma-related intrusions over the first three days. For vividness, heart rate measures from the scenes were significant predictors when entered in the second step (*p* < 0.05). Again, a higher heart rate during the assault sequence (*β*_AssaultScene_ = 0.505, *SE* = 5.10) predicted more vivid intrusions, while the opposite was found for the murder scene (*β*_MurderScene_ = − 0.552, *SE* = 5.13). When peritraumatic emotion ratings were included in the third step, only the heart rate for the murder scene remained significant, accounting for 25.8% of the variance in intrusion vividness (*p* = 0.163) (see supplementary Table S2). Comparable patterns emerged for the regression models predicting distress. Peritraumatic heart rate from the two scenes predicted later intrusion distress, following the same directional pattern (*β*_AssaultScene_ = 0.624, *SE* = 4.08; *β*_MurderScene_ = -0.633, *SE* = 4.10, *p* < 0.01). Inhibitory control and emotion ratings did not significantly contribute. The full model explained 36% of the variance (*p* < 0.05) (see supplementary Table S3). An overview of the predictors’ contribution to each full regression model is provided in Table 2.

**Table 2:** Overview of main results for all multiple regression models.

Criterion predictors	Intrusion frequency	Intrusion vividness	Intrusion distress	IES score	IES hyper-arousal	IES intrusion	IES avoidance	RTs negative reminders
Inhibitory control	0.043	− 0.004	− 0.056	0.312	0.082	0.195	0.418*	0.443*
HR assault	0.801**	0.430	0.479*	0.433	0.432*	0.283	0.343*	0.572*
HR murder	− 0.749**	− 0.507*	− 0.536*	− 0.110	–	–	–	− 0.251
Emotions assault	− 0.075	0.357	0.293	0.097	–	–	–	− 0.118
Emotions murder	− 0.079	− 0.161	− 0.039	− 0.051	–	–	–	0.022
Full model	R^2^ = 0.36	R^2^ = 0.26	R^2^ = 0.36	R^2^ = 0.26	R^2^ = 0.26	R^2^ = 0.13	R^2^ = 0.31	R^2^ = 0.37
	*p* = .038	*p* = .163	*p* = .038	*p* = .148	*p* = .046	*p* = .154	*p* = .006	*p* = .034

Standardized coefficients (beta) are displayed for the full model (step 3 in the hierarchical regression, see results for more details and supplementary material tables S1–S5 for all coefficients). Higher inhibitory control scores represent lower inhibitory control.

*p < .05; **p < *.*01.

### Inhibitory control and heart rate relate to subscales of the Impact of Event scale

The full model explained 26.5% of the variance in the IES score (*p* = 0.148), but only the second model, which included inhibitory control and peritraumatic heart rate, was significant (*p* < 0.05) (see supplementary Table S4). Participants’ inhibitory control (*β* = 0.326, *SE* = 1.92, *p* = 0.061) and the peritraumatic heart rate during the sexual assault scene showed marginal significance (*β* = 0.450, SE = 2.56, *p* = 0.052). Three multiple regression analyses were conducted on the IES subscales (hyperarousal, intrusion, and avoidance) using baseline inhibitory control and heart rate during the assault scene as predictors (see supplementary Table S4a–c).

For hyperarousal, the overall model was significant (*F*(2, 28) = 3.44, *p* < 0.05), with predictors explaining 26% of the variance. Peritraumatic heart rate significantly predicted hyperarousal (*β* = 0.432, *p* < 0.05), but inhibitory control did not (*β* = 0.082, *p* = 0.633). The model for the intrusion subscale was not significant (*F*(2, 28) = 2.00, *p* = 0.154). For the avoidance subscale, the model was significant (*F*(2, 28) = 6.29*, p* < 0.01), explaining 31% of the variance, with both inhibitory control (*β* = 0.418, *p* < 0.05) and heart rate (*β* = 0.343, *p* < 0.05) contributing significantly.

### Change score analyses

The complementary analyses using participants’ change scores as predictors, rather than the average heart rate across participants, broadly confirmed our main findings. The key difference was that the change score for the murder scene did not emerge as a significant predictor (see also supplementary Table S6).

### Task-irrelevant reminders impair cognitive interference resolution

We hypothesised that exposure to task-irrelevant trauma reminders would negatively affect performance on a demanding cognitive control task. To test this, we contrasted processing speed on the cognitive-interference task when incongruent stimuli associated with higher control demands were preceded by reminder or non-reminder images.

**Fig. 4:**
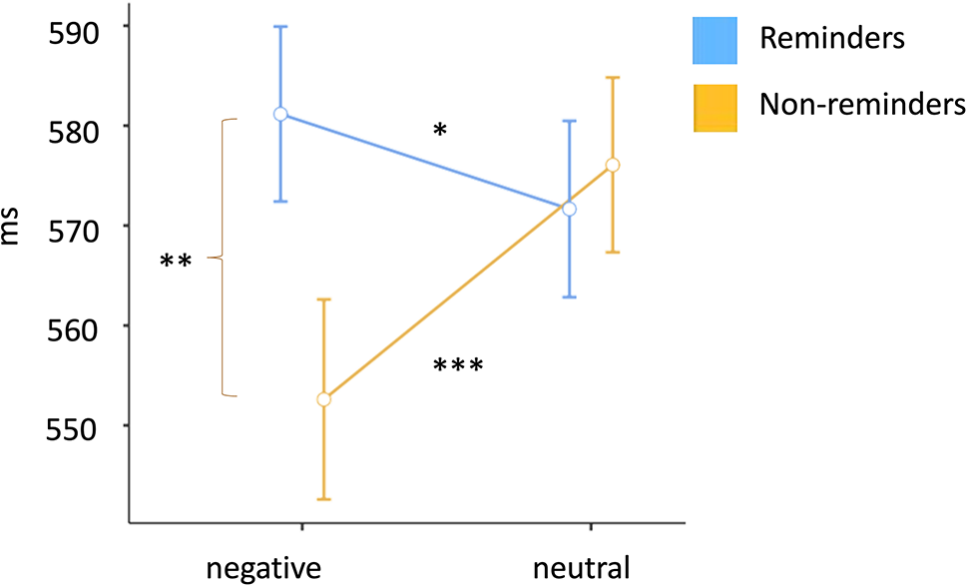
Reaction times for incongruent trials with primes grouped by valence. *Note*. The y-axis represents the mean reaction time scores (ms = milliseconds) for facilitating interpretation, though all analyses were done with log-transformed median reaction times per participant and condition. Error bars represent the standard errors. *p < .05; **p < .01; *** *p* < .001.

A 2 × 2 ANOVA comparing RTs for reminders and non-reminders with negative versus neutral revealed a main effect of *Prime* (reminder versus non-reminder), was revealed (*F*(1, 113) = 4.25,* p* = 0.042, η^2^ = 0.004), but no main effect of *Valence* (negative versus neutral). The interaction between Valence and Prime was significant, *F*(1, 113) = 9.97, *p* < 0.001, η^2^ = 0.007, indicating that negative reminders led to slower RTs (*M*_negative_reminder_ = 581 ms, *SD* = 93.5) compared to neutral reminders (*M*_neutral_reminders_ = 572 ms, *SD* = 94.2), *p* = 0.031 with a small effect size, *d* = 0.18, CI[− 0.009, 0.361]. For negative and neutral non-reminders, we did not predict a specific effect direction due to mixed findings in prior research, depending on the method, study design and sample^21^. However, the analysis showed that negative non-reminders yielded significantly faster RTs (*M*_negative_non-reminders_ = 553 ms, *SD* = 106.9) compared to their neutral non-reminders (*M*_neutral_non-reminders_ = 576 ms, *SD* = 93.4), *p* < 0.001, with a moderate effect size (*d* = 0.323, CI[0.134, 0.511]). Figure 4 depicts RT differences for reminders and non-reminders, moderated by valence.

### Inhibitory control and peritraumatic heart rate predict reminder interference

The results indicated that *negative reminders*, rather than reminders in general, caused significant response delays. Therefore, we used the median RTs for trials with negative trauma reminders as a proxy for participants’ ability to handle cognitive interference when reminded of a trauma-analogue scene.

The full regression model predicting RTs for trials with negative trauma reminders explained 37% of the variance (*p* < 0.05) (see Table S5). Significant predictors included baseline inhibitory control (*β* = 0.443, *SE* = 15.4, *p* < 0.05) and peritraumatic heart rate during the assault movie (*β* = 0.57, *SE* = 21.5, *p* < 0.05). Lower inhibitory control and higher heart rate during the sexual assault scene were associated with longer response times when encountering negative trauma reminders during the cognitive-interference task.

### Potential memory reactivation leads to a cognitive cost for the higher impacted group

Building on prior research analysing trauma survivor subgroups by symptom severity^27,28^, we conducted a median split of our sample's IES scores to explore variability in trauma-analogue symptom development. We hypothesised that perceived event impact would differentiate participants’ performance in handling negative reminders during the cognitive-interference task. Following previous findings showing RT delays for negative reminders, we calculated the ME by subtracting median RTs for accurate baseline trials preceded by negative reminders from those for incongruent trials per participant. A higher ME indicates stronger interference effects on processing speed^21^. Baseline conditions were used to quantify facilitation (congruent trials) and interference (incongruent trials).

**Fig. 5:**
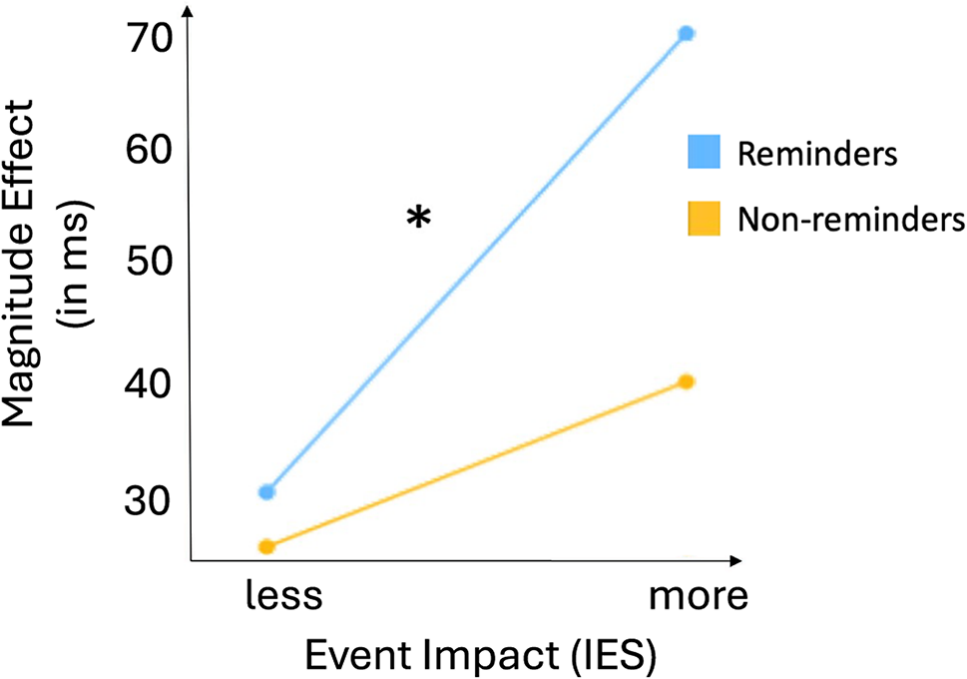
Mean Magnitude Effect for negative reminders versus negative non-reminders between groups with more versus less self-assessed event impact. *Note*. Event Impact measured with Impact of Event scale (IES), ms = milliseconds, * p < .05.

The more impacted IES group revealed a significantly higher ME (*M*_more_impact_ = 71.4 ms, *SD* = 67.8) for negative trauma reminders compared to the less impacted group (*M*_less_impact_ = 31 ms, *SD* = 38.0), *t*(29) = 1.98, *p* < 0.05, *M*_*diff*_ = 40.4 ms) with a moderate to large effect size (*d* = 0.72) (see Fig. 5). We compared MEs for negative non-reminders to rule out the effect being due to general performance differences rather than memory reactivation. Since no significant difference was found between the IES groups (*M*_more_impact_ = 40.9 ms, *SD* = 71.4; *M*_less_impact_ = 25.5, *SD* = 41.8; *t*(29) = 0.71, *p* = 0.483; *M*_*diff*_ = 15.4 ms), the effect likely stems from memory reactivation rather than general performance differences.

## Discussion

This study aimed to investigate factors that may explain the impact of a trauma-analogue experience on healthy individuals using a trauma film paradigm. Additionally, we tested the effects of incidental reactivation of trauma-like memories on cognitive interference resolution.

Our study showed that exposure to trauma-analogue film scenes elicited both vivid and distressing intrusive memories in participants, which diminished over a few days, a finding consistent with prior research employing the trauma film paradigm^11,18^. The frequency and quality of these intrusions were predicted by peritraumatic heart rate. A higher heart rate during the assault scene was positively associated with intrusion outcomes, whereas a lower heart rate during the murder scene was linked to more intrusions. These findings suggest that physiological responses during a trauma-analogue experience may serve as important indicators for the subsequent development of PTSS. The overall IES score, which assesses self-reported symptoms after a stressful event, was not significantly predicted by any of the examined factors. However, further analyses revealed that a higher heart rate during the assault scene predicted increased self-reported symptoms on both the hyperarousal and avoidance subscales. Additionally, lower inhibitory control was associated with greater avoidance symptoms. Conversely, neither of these factors predicted scores on the intrusion subscale of the IES.

In a task with high cognitive control demands, task-irrelevant negative trauma reminders led to slower responses than neutral reminders and non-reminders. This RT cost was predicted by both lower baseline inhibitory control and higher perifilm heart rate. Further subgroup analyses revealed that the interference effect was solely attributable to individuals who were comparably more impacted by the trauma analogue.

### Peritraumatic response predicting trauma-analogue symptom development

Increased mean heart rate during the sexual assault scene predicted the frequency, vividness and distress of intrusions, heightened hyperarousal and avoidance symptoms, as well as impaired reminder interference control. These findings imply heightened accessibility of the most negative trauma-analogue memories, which is consistent with previous research suggesting that increased physiological arousal during traumatic experiences^34,35^ could enhance the encoding and subsequent retrieval of traumatic memories. Conversely, the scene depicting a murder was accompanied by a decreased heart rate predicting intrusions. This may signify a maladaptive reaction, implying diminished emotional engagement and heightened susceptibility to dissociation^44,45^. It is conceivable that our mostly female sample identified more with the woman depicted as the rape victim, leading to higher physiological arousal. A numerical comparison of heart rate changes during the assault scene in contrast to the control scene, and split by gender, aligns with this suggestion (see supplementary material S7) but does not exclude the possibility that females in our sample generally reacted with higher arousal to the trauma-analogue content. In contrast, the violent male murder scene might have led to coping by dissociation. This interpretation remains speculative since dissociative responses were not measured in our study.

However, while the complementary analyses using change scores to account for inter-individual differences in heart rate responses broadly confirmed our main analyses, the murder scene did not emerge as a significant predictor of trauma-analogue symptoms. This may be due to the potentially nuanced nature of physiological responses to distressing stimuli. One possibility is that participants exhibited a blunted arousal response—a pattern observed in trauma-related dissociation or freezing reactions^66,67^. By reducing variability in the data, the change score approach may have failed to capture individual differences in physiological reactivity relevant for trauma-related symptom prediction, particularly if a blunted arousal response did not manifest as a significant change compared to our control condition. Exploring the possibility of a more nuanced autonomic nervous system response by analysing HRV measures across participants did not reveal any differences between either trauma-analogue scenes or the control condition. This null result aligns with a recent review concluding that heart rate and HRV measures yield mixed and inconclusive findings regarding dissociative responses^66^. However, it may also stem from the limited interpretability of our control condition or other less apparent methodological constraints.

Capturing the phenomenological experiences of trauma-analogue exposure has been proven a valuable indicator of reported impact and intrusion frequency in prior research^31,68^. However, while peritraumatic emotion ratings served as a manipulation check in our study, they did not demonstrate any predictive value, suggesting they may not be reliable indicators of trauma-related impact in our sample. A rating scale with predefined emotions may be too simplistic to capture variability in experienced distress, let alone a holistic phenomenological experience. The understanding of certain emotion words could also vary between individuals. A pre-validated measurement tool such as the *Peritraumatic Distress Inventory*^69^ may have been more suitable if adapted for the trauma film paradigm. Thus, future studies could consider utilizing more comprehensive and nuanced measures by including both pre-validated scales and open-ended inquiries capturing sensory, perceptual, and emotional aspects of peritraumatic experiences.

### Inhibitory control as a protective factor?

Contrary to our predictions, baseline inhibitory control did not predict the emergence of intrusions in our sample. Prior research has demonstrated an association between inhibitory control measures and symptom severity. Yet, these studies have not explicitly examined the relationship with intrusion frequency, instead focusing more broadly on general symptom severity or the presence versus absence of a PTSD diagnosis^27,28^. Studying real-life trauma presents limitations in understanding whether inhibitory control functions as a preceding protective mechanism or was enhanced in the aftermath due to the recurrent need to manage trauma-related thoughts, potentially leading to plastic changes in inhibitory control^70^. Therefore, we examined whether baseline inhibitory control would predict subsequent event impact and based on previous work employing a trauma film paradigm showing that reduced memory control was related to increased intrusions^12^. While inhibitory control is believed to moderate the ability to suppress unwanted thoughts and thus play a beneficial role in maintaining mental health after trauma exposure^23,71^, the simple Colour-Word Stroop task used as a baseline measure might not capture the intentional thought control facet of memory inhibition.

Our results indicate that reduced inhibitory control and heightened peritraumatic heart rate predicted elevated avoidance behaviour one-week post-exposure. The avoidance subscale assesses the tendency to avoid or suppress distressing or intrusive memories, emotions, or cues related to the traumatic experience, with higher scores indicating greater efforts to distance oneself from the psychological impact of the traumatic event^47,72^. Consistent with this interpretation, lower baseline inhibition was also associated with more interference from trauma-related reminders, suggesting a heightened cognitive processing burden accompanying the unintentional reactivation of trauma memories.

Thus, inhibitory control in our sample might not be as connected to the degree of intrusive memories as to the successful effort to handle interference from upcoming trauma reminders. Early interventions following trauma exposure may target improving individuals’ capacity to cope with unwanted reminders by effectively deploying inhibitory control. Future studies should consider measuring individuals’ ability to target thought and memory control, such as with the Think No Think task^73^, to elucidate the relationship between inhibitory control and intrusion development.

### Susceptibility to trauma reminders as a measure for trauma-analogue impact

Patients suffering from PTSD are easily triggered by reminder cues, which can lead to the reliving of a sensory-rich aspect of their original trauma^48^. We sought to capture variability in the expression of this symptom within a healthy population, thereby complementing recent clinical investigations^22^. Our approach leveraged a novel paradigm to indirectly measure cognitive interference caused by trauma reminders through response time differences. However, trauma reminders displaying inherently neutral scenes (e.g., the sign of a nightclub featured in one of the scenes) did not cause a response time delay, which could have been expected if a related cue had triggered a trauma-like and highly accessible memory. However, the carefully selected sample and the comparably mild stressor elicited by trauma films may be the reasons for not observing memory reactivation as for the inherently negative reminder cues.

Our finding that negative trauma reminders caused delayed processing speed for the group of participants more impacted by the trauma analogues indicates the incidental reactivation of related trauma-like memory, leading to an increased burden on the cognitive-interference task. The observed effects imply a subtle degree of maladaptive memory reactivation in healthy individuals who experienced more negative impact by the trauma analogue. Though the subgroups in our sample were small, the findings indicate we captured meaningful variability in trauma-analogue impact with both subjective (IES) and objective measures (RTs). These effects may become more pronounced following exposure to a genuine traumatic event, potentially contributing to cognitive impairments and disruptions in daily life for those most severely affected, consistent with the experiences of individuals diagnosed with PTSD^11,23^. The use of response latencies as indicators of reminder interference might be a meaningful measure of the degree of individual trauma impact and susceptibility to further symptom development. The connection between subjective event impact and maladaptive reminder interference may be reciprocal. Specifically, an increased propensity for maladaptive memory reactivation could reinforce the overall development of PTSS. Cognitive models of PTSD emphasise intrusions and, thus, vulnerability to trauma reminders as a critical symptom of the disorder, which may then elicit other symptoms within the broader symptom network^11^. Although beyond the scope of the current investigation, future longitudinal studies should consider causal modelling approaches to elucidate the assumed complex relationships among the various symptom clusters of PTSD.

Collectively, our results complement recent research, showing the impact of potential trauma-related memory reactivation on response time delays^22^. They also extend clinical research that relates variability in PTSS to potential risk and resilience factors^27,28,74^ to a non-clinical sample.

### Strengths and limitations

Our study employed a film paradigm to manipulate traumatic material in a safe, controlled environment, inducing trauma-analogue symptoms that share core features with real trauma exposure^75^. However, caution is needed before generalising these findings to populations exposed to genuine trauma. While the selected trauma-analogue scenes have been validated in prior research^18,37^, two-thirds of post-exposure intrusions were related to the sexual assault scene. Both scenes were perceived as more distressing than the neutral control scene, but their impacts differed. Furthermore, our study could have benefitted from capturing potential dissociative responses which might have allowed us to illuminate the observed opposite effects of heart rate depending on the trauma-analogue scene.

Interestingly, none of our predictors in the main analyses explained variability in the IES *intrusion subscale*, despite our finding that heart rate predicted both frequency and quality of self-reported intrusions. This may reflect a limitation of trauma-analogue paradigms, in which the impact of the stressor diminishes over a short period. When participants completed the IES one week post-exposure, intrusive symptoms may have subsided, whereas our daily intrusion logs provided a more immediate and sensitive measure. Additionally, the IES-R Intrusion subscale includes items related to nightmares and sleep disturbances, which were not reported in our sample.

The final sample size was limited due to the exclusion of many potential participants (90 pre-screenings, 40 met inclusion criteria, 32 completed the study). Thus, the sample comprised a carefully screened group, and its homogeneity may be considered a strength. Still, observed effects in this healthy, preselected group likely underestimate true effect sizes in more vulnerable populations. The overrepresentation of female participants in our sample presents a limitation that may affect the generalizability of our findings, particularly in light of potential gender differences in physiological and emotional responses to the trauma-analogue material. Our results may primarily reflect patterns characteristic of female participants and should not be assumed to generalize across genders. Moreover, expanding trauma-analogue material to include less gendered content would help assess whether observed effects are stimulus-specific or more broadly applicable. Future research should aim to include more gender-balanced samples to explore possible gender-specific effects more thoroughly. Another limitation of our study relates to the choice of control scene (see supplementary materials). While selecting a control scene from the same movie ensured consistency in cinematography, the presence of the same actors as in the trauma scenes may have introduced transfer effects. However, post-scene ratings confirmed that participants perceived the control scene as neutral compared to the trauma scenes. Notably, while subjective ratings indicated that the murder scene was more distressing than the control scene, this difference was not reflected in heart rate responses, suggesting a dissociation between physiological and subjective measures for this particular comparison. Future studies may benefit from employing control scenes from entirely different sources to further mitigate potential confounds.

Based on established theory and prior empirical research, we measured autonomic nervous system responses and executive functioning to explore individual differences in trauma-analogue symptom development. The limited sample size precluded examining other factors like genetics, personality, or neuroimaging, which remain important avenues for future research. Future studies could employ memory reactivation paradigms to assess symptom changes before and after therapeutic interventions. This approach would benefit from involving ecologically valid designs that utilise personally relevant reminder cues from individuals’ autobiographical memories with varying levels of traumatic content. By incorporating both involuntary and voluntary retrieval paradigms, such studies could examine potential changes in memory accessibility, phenomenology, and dual-task performance while tracking neural and physiological responses. Targeted interventions to improve inhibitory control may reduce symptoms by helping individuals cope with or suppress trauma reminders. Additionally, further research on neural correlates of reminder interference and trauma-memory reactivation could shed light on the mechanisms underlying trauma susceptibility and symptom change.

## Conclusions

The results of this study shed light on the intricate interplay of various factors relating to the degree and consequences of trauma-analogue impact in a non-clinical sample. We were able to predict different facets of the adverse effects of trauma-analogue exposure, including intrusion frequency and quality, self-assessed event impact, and the efficiency of handling trauma reminders through measures of peritraumatic heart rate and inhibitory control. However, these predictors were relevant to trauma-analogue symptoms to different extents, with peritraumatic heart rate primarily associated with intrusion characteristics, while inhibitory control played a role in managing trauma reminders and avoidance behaviours. Overall, the findings suggest that inhibitory control and peritraumatic heart rate serve as relevant predictors of trauma-analogue symptoms. Furthermore, reminder interference may prove to be a meaningful diagnostic measure for assessing trauma-related impact and the risk of PTSD development.

## Supplementary Information

Supplementary Information.

## Data Availability

The data that support the findings of this study are not openly available but are available from the corresponding author upon reasonable request.
