# Supplementary material for: Trauma-analogue symptom variability predicted by inhibitory control and peritraumatic heart rate
Source: Sci Rep. 2025 Apr 30;15:15215. doi: 10.1038/s41598-025-99564-x (PMC12044069; doi:10.1038/s41598-025-99564-x)
Supplement: Supplementary file 1 — Supplementary Information. [file 41598_2025_99564_MOESM1_ESM.docx]

***Trauma-analogue symptom variability predicted by inhibitory control and peritraumatic heart rate -* Supplementary Material**

**Pre-screening process & final sample**

Due to the likely emotionally distressing nature of the trauma-analogue study, participants were carefully recruited digitally and in-person to take part in a “movie study” through a pre-screening process. Interested participants received an informational email which included a detailed explanation of the experiment and link to a secure psychological screening. The survey was constructed according to previous research that implemented the trauma film paradigm to ensure that unsuitable and potentially vulnerable individuals were excluded ^1^. Exclusion criteria included mood altering medication, drug abuse, uncorrected visual or hearing impairment, neurological or mental disorders, depression symptoms assessed with the PHQ-9 (score > 4) ^2^, anxiety symptoms assessed with the STAI trait scale (> 44 points) ^3^, and trauma exposure needing psychiatric care. Individuals who were under 18 years of age or had previously seen the experimental film were also excluded. 
 The pre-screening process was supervised by a clinical psychologist and each survey was manually reviewed for mental health and trauma history. After review, all submissions received a response via email notifying individuals of their eligibility or ineligibility to participate. In total, 90 identifiable and complete pre-screenings were considered after submission, 40 individuals met inclusion criteria, and 32 individuals completed the study. In line with similar studies drawing on a within-subject design, the target number of participants was set to a minimum of N = 30 ^4^.

**Laboratory and materials**
 The experiment was conducted at Lund University’s Humanities Lab, Lund, Sweden. The lab computer ran on Windows 10 Pro version 1909 and the experimental movies were watched on a 22 inch 59 Hz Dell P2210 flat panel monitor with resolution 1680 x 1050. A set of Behringer HPS3000 headphones were used to provide audio to the participants. The volume of the audio was standardized at 70% for all participants. The movie scenes, inhibitory control and cognitive-interference tasks were presented using the E-Prime 3.0 software ^5^. Go/no-go task responses were collected on a Dell keyboard and cognitive-interference task responses were recorded on a Chronos multifunctional response and stimulus device.

**Psychophysiological Measurements** During the trauma-analogue film exposure and the subsequent cognitive-interference task 24-48 hours post trauma-analogue exposure, we measured participants’ electrocardiogram (ECG) and galvanic skin response (GSR). The software AcqKnowledge 5.0 ^6^ was used for the acquisition. A MP150 system with a ECG100C unit was used to record the electrocardiogram of participants. Leads 100-S and disposable electrodes EL501 were used to connect participants to the ECG100C unit with electrodes connected to both wrists and the neck. All the equipment described was manufactured by BioPac systems ^6^.
 The GSR100C unit and ECG100C unit were used to record electrodermal activity and electrocardiogram (ECG) respectively. Another sub-aim of this study was piloting both the paradigm and testing its suitability for a planned fMRI study. However, while the GSR measurements of physiological activity as an indication of heightened arousal would have been of interest for the main research question of this study, faulty lab material, (i.e., the applied electrode gel for skin conductance), made a reasonable analysis impossible which led us to disregard the collected GSR data altogether.

**Inhibitory Control Tasks**
 The Stroop Colour and Word Test contained a series of three displays which the participants were required to read through aloud. The first presentation was an 8x6 block of coloured dots, the second was an 8x6 block listing the names of colours in black ink, and the final presentation was the Stroop test condition containing an 8x6 block of incongruently coloured words where participants had to name the colour of the ink, not read out the word. For example, “red” was listed in blue ink and the correct response was “blue”. 
 The Go/NoGo was designed with letter stimuli presented on a white screen where participants were instructed to press ‘space’ (go trials) for quickly appearing letters except the letter ‘X’ (no go trials). The task was designed so that the ‘go’ to ‘no go’ trial proportion was an 80/20 ratio totalling two blocks of 100 trials each. This ratio served to initiate prepotent motor response.

**Film scenes**
 The trauma-analogue exposure consisted of four, 9-minute clips from two movies totalling 36 minutes of run time. Three separate scenes from the film *Irréversible* ^7^ and one scene from the film *Paterson*^8^ were selected for the experiment, two trauma-analogue and two non-traumatic scenes. The movie stimuli were alternated so that participants were always shown a nontraumatic then a trauma-analogue scene. The order of trauma-analogue scenes was counterbalanced across participants while the scene from the movie *Paterson* was always the first scene participants viewed before the first trauma-analogue scene. The *Paterson* scene was a mundane portrayal of a bus driver’s day. We disregarded that scene as a potential additional baseline for subsequent analyses as several participants reported after the study that the trigger warning which was read out to participants just before the start of the first clip raised apprehensive expectations that a traumatic scene would follow. The two trauma-analogue scenes selected from the movie *Irréversible* portrayed a sexual assault and a murder, respectively. The control scene was taken from the same movie *(Irréversible)* to control for lightning, camera work and actors. The scene depicted a mundane commute in a metro with the three main characters engaging into small talk.

**The cognitive-interference task (T2)**

The cognitive-interference task utilized a block design wherein reminder and non-reminder images, negative and neutral stimuli were separated by blocks, not trials. Accounting for images from all four films and control images, a total of 120 unique distractor images were shown in the cognitive-interference task. Thirty images were taken from each *Irréversible* film clip, 15 of which were neutral in valence and 15 were negative in valence. Fifteen images were taken from the mundane *Paterson* film which was only neutral in valence. Thirty images were taken from the Nencki Affective Picture System^9^ (NAPS) research database which included 15 neutral images and 15 negative images, varying in depicting scenes of animals, humans, body parts and objects. For example, neutral images included pictures of buildings, non-threatening animals, or fruits, while negatively rated images featured depictions of mutilated bodies, crime scenes, or injured animals.The movie screenshots were chosen by the experimenters and went through a multi-step selection process in order to most likely capture trauma hotspots (negative valence) and neutral reminders without traumatic content. During the piloting, participants were asked to rate the valence of the selected images from the trauma movies in order to optimize face value selection. This piloting was done with participants (N = 3) who were ignorant to the movie and would judge the valence only based on the content of the images seen. Only images of our initial pre-selection with a negative value of at least 3 (“You are judging this image as ...” from 1 (very negative) to 9 (very positive) and between 4-6 for the neutral ones would be included into the study. All screenshots and images were sized to 1920 ×1080 pixels for optimal resolution.

In total, the task was comprised of 320 pseudo-randomized trials including one practice block of 20 trials (no distractor image).The practice block was followed by 16 experimental blocks of 20 trials each. Every trial was preceded by a 1,5 second fixation cross, distractor images were then presented for 3 seconds, followed by a short 500ms fixation cross and the respective Stroop trial (500ms) and a 500ms extended response window. Participants were able to respond to the trial with onset of the same, giving a total of 1 second response window. A trial could vary in cognitive demand, i.e., congruent, baseline or incongruent. For example, an incongruent trial design would present the participant with “444” and it was their job to press the corresponding button on the response box (numbered 1-4) that represented the correct number of characters, not the number itself. For a congruent trial the number of characters would match the shown number (e.g., “333”) while a baseline trial would require participants to count one to maximal four hashtag symbols.

**Data Preprocessing**

***Inhibitory Control Scores – Stroop & Go/NoGo*** The Go/NoGo score was derived from a hit rate – false alarm rate accuracy formula ^10^. The Stroop Color and Word Test score was calculated using a formula which accounted for reaction time in both baseline and incongruent conditions (incongruent – [(coloured dots + black words)/2]) ^11^ with higher scores indicating lower inhibitory control capacity.

***Intrusion frequency & quality*** Memory intrusion frequency was measured on a scale from 0 to 4+ and participants reported them for six consecutive days. Total intrusion frequency was calculated by adding up the individual scores per participant and day. Vividness and distress potentially caused by the intrusions were rated from 0-100 and averaged separately per participant over the course of the first three days following trauma-analogue exposure at T1.
***Impact of Event scale – revised*** A minimum of 0 and a maximum of 100 points could be scored reflecting the self-assessed the trauma-analogue symptoms on day seven after trauma-analogue exposure (T1). The total IES score was calculated per participant as well as for the Intrusion, Avoidance and Hyperarousal subscales respectively. Subscale scores were calculated by taking the mean from the respective items on that score, following instructions of the scale manual ^12^.
***ECG recoding and processing*** The ECG data was recorded at a sampling frequency of 2000 samples per second. The signal underwent visual inspection for major artifacts using AcqKnowledge, which involved generating a raw tachogram. One dataset was excluded due to significant artifacts. Further, we disregarded our baseline collected during the initial rest period as a reliable comparison because of problems with the electrode adherence affecting around half the sample which resulted in suboptimal short baseline measures for around 40% of the sample. Before the experiment commenced, participants were repositioned towards the screen, their headphones were adjusted, and they were instructed on proper hand placement to minimize measurement interference. At this stage, electrode positioning and adherence were systematically checked, which led to enhanced signal quality from the outset of the experimental phase. Subsequently, the raw ECG signal was processed using a MATLAB script with assistance from the recommended HRV Toolbox developed by M. Vollmer ^13^. To analyse heart rate, an algorithm for automated heartbeat detection was employed to extract RR data from the annotation data for each of the film scenes per participant. RR data represents the time intervals between successive R-peaks in the ECG, where R-peaks signify the highest peaks in the QRS complex. The QRS complex, consisting of the Q, R, and S waves, reflects the ventricular depolarization of the heart in the ECG signal. To enhance the quality of the RR data, a bandpass filter with a 0.5 to 40 Hz cut-off frequency was applied and additional artefacts were automatically removed based on the z-score of the RR interval. Furthermore, remaining outliers were detected by excluding data points beyond determined cut-off values obtained by adjusting 2.5 times the interquartile range around the first and third quartiles. Data points outside this range were identified as outliers and removed from the final dataset. Outlier detection was performed separately for each time series corresponding to individual video segments. The heart rate per participant during the distinct film scenes was calculated based on the filtered RR intervals using the heart rate function from the HRV Toolbox ^13^.

Heart rate variability (HRV) was analyzed using M. Vollmer’s HRVTool in MATLAB, based on filtered RR interval data derived from electrocardiogram (ECG) recordings. Prior to analysis, RR intervals were preprocessed to remove artifacts and ectopic beats using an adaptive filtering approach. Time-domain measures, including SDNN (standard deviation of NN intervals), RMSSD (root mean square of successive differences), and PNN50 (percentage of successive NN intervals differing by more than 50 ms), were computed to assess overall HRV and parasympathetic activity. For frequency-domain analysis, a Fourier transformation was applied to extract low-frequency (LF, 0.04–0.15 Hz) and high-frequency (HF, 0.15–0.40 Hz) power components, reflecting autonomic nervous system dynamics. The LF/HF ratio was calculated as an indicator of sympathovagal balance.
***Outlier detection for the behavioural data analyses*** Before analyses, the sample was tested for potentially influential outliers with visual inspection of box plots and additionally identification by calculating [Q1 - / Q3+ 1.5 * IQR] (Q1 = 25^th^ quartile, Q3 = 75^th^ quartile, IQR = interquartile range). We chose a conservative approach in order to keep statistical robustness as high as possible and only excluded one outlier which showed substantial impact on our statistical results. This participant was identified as an extreme outlier on two of our dependent variables, scoring the maximum on both IES and intrusion vividness across the sample which showed high leverage on our data.

**A priori results**

***Validation of Selected Time Windows: Heart Rate Differences Between Scene Segments***
 In order to isolate the trauma hotspots in our scenes and additionally control for potential general initial arousal / apprehension effects not related to the respective scene content, we chose to analyse the hotspots to the middle / later part of the scenes for the respective trauma-analogue scene (see also p. 9 in the main manuscript) and a 2 min section from the middle of the control scene. As a control analysis to check if the parts we chose would differ from their beginning (first 2 min of the respective scene) we conducted 3 paired sample t-tests, comparing the mean heart rate of the first2 min and the selected 2 min within the movie scene.

For the murder scene, there was no significant difference in heart rate between the beginning (M = 74.6, SD = 7.16) and the hotspot (M = 73.6, SD = 7.04), t(29) = 1.16, *p* = .257, d = 0.21). For the assault scene, heart rate was significantly higher for the hotspot (M = 78.9, SD = 10.43) compared to the beginning (M = 74.6, SD = 7.76), t(29) = -2.69, *p* = .012, d = 0.49). For the control scene, there was no significant difference between the beginning (M = 74.4, SD = 7.58) and the middle (M = 74.6, SD = 7.00), t(29) = -0.26, *p* = .797, d = 0.05.

These results suggest that heart rate stayed stable for the control scene which is indicating that taking the middle part or the beginning of the scene would not have made a difference for our main analyses. For the trauma-analogue scenes we find no change for the start and hotspot of the murder scene and an increase from the beginning to the hotspot for the assault scene. Given the differing narrative structures of the scenes, direct comparisons are challenging. The murder scene begins already with intense music and chaotic action, culminating in a violent confrontation and eventually brutal killing of the main character (the hotspot), whereas the assault scene starts with a calmer sequence of a woman entering a metro tunnel coming from a social event before more slowly escalating until the hotspot (the rape). This contrast supports the rationale for analyzing the main hotspot within each scene rather than averaging HR across the entire scene or using identical time windows across scenes.

***Both trauma-analogue scenes were distressing, but with different effects on heart rate*** We expected based on the majority of prior research to find an increase in physiological arousal as well as the experience of increased negative emotion ratings for the trauma movies compared to the control condition. To check our assumptions, we ran a comparison between heart rate for the respective trauma scenes versus the control scene. The same was applied for the peritraumatic emotion ratings.
 Comparing the heart rate of the sexual assault scene with the control scene confirmed our expectation of observing higher heart rate for the trauma scene (t = -1.94, *p* = .028, d = .49; M_assault_ = 78.8; SD_assault_ = 10.2 ; M_control_ = 74.5; SD_control_ = 6.79). However, the heart rate for the two-minute section picked from the murder scene showed a numerical *decrease* in mean heart rate compared to the control scene (t = 0.649, p = .259, d = 0.165; M_murder_= 73.3; SD_murder_ = 6.80). For the peritraumatic emotion ratings, the initially eight emotion ratings were submitted to a Principal Component Analysis (PCA) to account for an underlying structure which could be meaningfully grouped together. The PCAs were done with the promax rotation method in order to account for the assumed correlational structure (opposed to prioritizing orthogonality). For the emotion ratings of the murder scene six emotions loaded high (>0.5) on one component (fear, disgust, anger, sadness, discomfort, surprise) while for the assault scene five emotions remained (fear, disgust, anger, sadness, discomfort) as one component. These were averaged into one peritraumatic emotion rating separately per scene and participant (see table also Table 1 in main manuscript).
 We compared the respective emotion ratings with the ratings for the control scene (using the respective counterparts for the assault and murder video). One sided t-tests showed significantly higher negative emotion ratings for the assault scene (t = -14.7, *p* < .001, d = 3.67; M_assault_ = 8.38, SD_assault_ = 1.32; M_control_ = 2.16, SD_control_ = 2) and the murder scene t = 8.50, *p*  <.001, *d* = 2.12; M_murder_= 5.97, SD_murder_ = 1.91; M_control_ = 2.11, SD_control_ = 1.72) compared to the respectively averaged emotion rating for the control scene.
 In conclusion, our pre-assumptions were mostly met apart from the numerically but not statistically *lower* heart rate for the exposure time of the murder video. Since the emotion ratings indicated a subjectively higher negative experience compared to the control video and prior research furthermore entertaining the idea that also *decreases* in heart rate could be meaningfully related to trauma symptoms ^14–16^ we kept the peritraumatic response (heart rate and emotion ratings) as predictors for both scenes but treated them as separate predictors.

***Exploring HRV measures across movie scenes***

Heart rate variability (HRV) measures can help clarify whether the observed heart rate differences reflect more subtle responses potentially connected to blunted arousal as a consequence of dissociation, providing insight into autonomic regulation and the balance between sympathetic and parasympathetic activity. Dissociation is linked to hypoarousal, where the initial passive reaction to a threat may coincide with a blunted autonomic response, however a recent review comparing studies with psychophysiological correlates of dissociative experiencing in PTSD patients was unable to provide robust evidence that peri- and post-traumatic dissociation are associated with specific effects of HRV ^17^ .

We first analysed RMSSD (Root Mean Square of Successive Differences) reflecting short-term variations, primarily parasympathetic (vagal) activity. Studies suggest that RMSSD can be reliably measured in short recordings ^18^. Other HRV measures such as SDNN, low frequency (LF), high frequency (HF) their ratio (LF/HF) and pNN50 have been shown to be more reliably over longer time frames > 5 min to hours which is why we analysed these respective measures for the whole 9 min of movie scene exposure ^19^ instead of taking the 2 min sections.

In summary across all heart rate variability (HRV) measures, within-subjects ANOVAs revealed no significant effects between the scenes. The specific coefficients of the analyses and descriptives are displayed in Tables S8 and S9.

***The trauma-analogues provoked vivid and distressing intrusions which decayed over time*** In order to follow up our research question concerning factors predicting the frequency and quality of intrusions, we first aimed to explore their occurrence and temporal development. As intended by the paradigm, intrusive memories of the trauma-analogue movie scenes were provoked in almost all participants with highest frequencies during the first three days following trauma-analogue exposure. The highest reported individual total over six days follow up were 21 separate intrusions while two participants reported none. Participants reported high vividness and distress caused by the respective intrusions after T1, descending almost consistently over the following week, reflecting a decline curve similar to the development of intrusion frequency. Since participants’ response behaviour on intrusion quality measures were most consistent during the first three days and over two thirds of intrusions were reported during this time, we averaged vividness and distress ratings separately per participant as potentially most representative for the quality of intrusions following exposure for these respective days.

***Cognitive inhibition and response inhibition did not correlate*** Recent research has suggested the domain-general nature of inhibitory control ^20,21^. These lines of research show not only that both response and cognitive inhibition share the same areas in the prefrontal cortex accounting for both response and thought inhibition^20^ but may also correlate in behavioural performance. However, a recent preprint concluded that thought control, especially retrieval stopping recruits a specific neural pathway, distinct from action stopping ^22^. Based on the ongoing discussion of domain-general versus domain-specific inhibitory control, we decided to employ both a cognitive and a response inhibition task. To test if we would find support from our behavioural findings for a domain-general account of inhibitory control ^20^ we ran a correlation analysis on the Go/NoGo and Stroop score. Since in our data response inhibition and cognitive inhibition showed no significant relation (Person’s r = -0.084, *p* = .653, df = 29), we treated them as two separate indicators capturing distinct aspects of inhibitory control. Since the Color Word Stroop as potentially closer to reflect the cognitive component of inhibition, has been shown to distinguish symptom pressure in individuals who had experienced trauma ^23,24^ we included only participants’ Stroop score into the subsequent analyses as a predictor.

***Varying cognitive control demand modulated response latencies***

As a manipulation check we tested with an ANOVA whether the RTs of the cognitive-interference task differed as expected between task conditions (i.e., congruent, baseline and incongruent). The overall model was significant, F(2, 6871) = 133, *p* < .001, indicating a significant effect of condition on participants’ RTs (M_incongruent_ = 597 ms; M_baseline_ =553 ms, M_congruent_ = 525 ms). Participants in the incongruent condition showed significantly slower RTs compared to the congruent condition (mean difference = −72.4 ms, t = −16.21, *p* < .001, d = −0.481) as well as compared to the baseline condition (mean difference = 44.6 ms, t = 9.98, *p* < .001, d = 0.297). As expected RTs were also significantly slower in the baseline condition compared to the congruent condition (mean difference = −27.8 ms, t = −6.30, *p* < .001, d = −0.185). The findings suggest that the condition of the cognitive interference task trials significantly affected RTs, demonstrating the classic Stroop interference effect ^11^ .

***Event Impact related to intrusion frequency***
 In order to further explore the assumption that our measures would meaningfully capture variability in event impact beyond the subjective IES ratings, we compared individuals with higher IES to those with lower IES scores on intrusion. Therefore, we conducted a median split on our sample’s IES scores, dividing them into the group of less impacted individuals (N = 14, M = 3.87, SD = 2.77) and more impacted individuals (N = 17, M = 20.6, SD = 10.5). We hypothesized that the more impacted group would have reported more intrusions compared to the less impacted group. Indeed, the more impacted group was found to experience significantly more intrusions (M_more_ = 8.79, SD = 5.49) than the less impacted group (M_less_ = 4.89, SD = 3.52), t(29) = 2.30, *p =* .015 *, M_diff_* = 3.90, 95% CI [1.01, Inf] with a large effect size of *d* = 0.83.

Alternative correlational approach:

Since we predicted based on the theoretical assumption that the number of experienced intrusions would meaningfully relate to a higher subjective rating of being negatively impacted by the trauma analogue, we explored if the IES score would be correlated with intrusion frequency. Indeed, the total intrusions and participants IES scores correlated significantly when tested in a one-sided Pearson correlation (r = 0.405, *p* = .012, df = 29).

***Change score analyses***

As complementary analyses to our main analyses and in order to isolate trauma-analogue scene related variation in participants’ heart rate, we used inter-individual change scores as predictor instead of mean heart rate per scene (*d’=* perifilm heart rate during trauma-analogue scene – control heart rate).

We ran all regression models as outlined in the main results but with the respective change scores as predictors. An overview of the results is displayed in Table S6.

For intrusion frequency, only the change score of the assault scene was a significant predictor (*p* < .05), for intrusion vividness, the model did not yield significant effects. However, for intrusion distress, the change score for the assault score was a significant predictor (*β* = .478, *p* < .05), and the model explained 32% of the variance.

Regarding the IES total scores, the regression model did not reach significance (R² = .25, *p* = .191). However, for IES hyper-arousal, the change score for the assault score significantly predicted self-assessed symptom severity (*β* = .383, *p* < .05), accounting for 16% of the variance (R² = .16, *p* = .091). Similarly, for IES intrusion symptoms, the change score for the assault scene remained a significant predictor (*β* = .36, *p* < .05), with the model explaining 17% of the variance. The model predicting the third subscale, IES avoidance, was significant (R² = .25, *p* = .019), with inhibitory control (*β* = .423, *p* < .05) being a significant predictor.

Finally, for reaction times to negative reminders, both inhibitory control (*β* = .41, p < .05) and the change score for the assault scene (*β* = .572, *p* < .05) significantly predicted response times, with the model accounting for 28% of the variance. Thus, these complementary analyses broadly confirmed the main analyses (see Results in manuscript) with the expection that the HR of the murder scene did not emerge as a significant predictor.

***Difference in heart rate change split by gender***

To explore the possibility that females identified stronger with the victim in the assault scene, leading to higher physiological arousal, we compared the mean change scores from the control scene to the assault scene, stratified by gender.

The small number of only n = 8 males for which we had valid ECG data excluded further analyses due to a lack of statistical power. However, we observed a numerical difference between males and females in that males had an average HR change from the control to the assault scene of Change_male_ = -1.75 (BPM) while females showed an increase in HR from the control movie of Change_female_ = 6.65 (BPM).

Interestingly, we also observed a heart rate decrease in males for the murder versus control scene, whereas females showed relatively stable heart rates. However, these findings should be interpreted with caution, as the control scene, as noted earlier and in the main article, was not optimally selected. In table S7 the change scores split by gender for the two different movie scenes are displayed.

**Regression Results**

**Table S1** *Multiple Hierarchical Regression results for Intrusion Frequency*

|  | B | SE B | *β* | *p* |
| --- | --- | --- | --- | --- |
| Dependent Variable: Intrusion Frequency  Step 1: *R_adj_^2^ = -0.034, p* = 0.914, *df1* = 1, *df2* = 29 | | | | |
| Intercept | 7.026 | 0.919 |  |  |
| IC | -0.102 | 0.935 | -0.0203 | 0.914 |
| Step 2: Δ*R^2^ = 0.343, p* = 0.009, *df1* = 3, *df2* =27 | | | | |
| Intercept | 7.026 | 0.772 |  |  |
| IC | 0.085 | 0.791 | 0.017 | 0.915 |
| HR murder | -3.521 | 1.055 | -0.669 | 0.002 |
| HR assault | 3.678 | 1.050 | 0.730 | 0.002 |
| Step 3: Δ*R^2^ =* 0.0167*, p* = 0.038, *df1* = 5, *df2* = 25 | | | | |
| Intercept | 7.026 | 0.792 |  |  |
| IC | 0.217 | 0.832 | 0.043 | 0.796 |
| HR murder | -3.769 | 1.128 | -0.749 | 0.002 |
| HR assault | 4.033 | 1.165 | 0.801 | 0.002 |
| Pt emo murder | -0.401 | 1.123 | -0.079 | 0.724 |
| Pt emo assault | -0.375 | 1.122 | -0.075 | 0.741 |

*Note.* All predictor variables are z-standardized.
IC = Inhibitory control, measured at baseline with the Stroop task
Pt emo = peritraumatic emotion rating of respective trauma-analogue movie scenes
HR = heart rate
B = unstandardized estimate, β = standardized estimate, p-value was set to alpha 0.05

**Table S2** *Multiple Hierarchical Regression results for Intrusion Vividness*

|  | B | SE B | *β* | *p* |
| --- | --- | --- | --- | --- |
| Dependent Variable: Intrusion Vividness  Step 1: *R_adj_^2^ = -0.034, p* = 0.929, *df1* = 1, *df2* = 29 | | | | |
| Intercept | 28.516 | 4.02 |  |  |
| IC | 0.369 | 0.09 | 0.0168 | 0.929 |
| Step 2: Δ*R^2^ = 0.185, p* = 0.124, *df1* = 3, *df2* =27 | | | | |
| Intercept | 28.52 | 3.75 |  |  |
| IC | 1.11 | 3.84 | 0.051 | 0.774 |
| HR murder | -12.16 | 5.13 | -0.552 | 0.025 |
| HR assault | 11.11 | 5.10 | 0.505 | 0.038 |
| Step 3: Δ*R^2^ =* 0.069 *, p* = 0.163, *df1* = 5, *df2* = 25 | | | | |
| Intercept | 28.516 | 3.73 |  |  |
| IC | -0.088 | 3.92 | -0.004 | 0.982 |
| HR murder | -11.518 | 5.31 | -0.507 | 0.046 |
| HR assault | 9.4672 | 5.49 | 0.430 | 0.097 |
| Pt emo murder | -3.550 | 5.29 | -0.161 | 0.508 |
| Pt emo assault | -7.845 | 5.28 | 0.357 | 0.150 |

*Note.* All predictor variables are z-standardized.
IC = Inhibitory control, measured at baseline with the Stroop task
Pt emo = peritraumatic emotion rating of respective trauma-analogue movie scenes
HR = heart rate
B = unstandardized estimate, β = standardized estimate, p-value was set to alpha 0.05

**Table S3** *Multiple Hierarchical Regression results for Intrusion Distress*

|  | B | SE B | *β* | *p* |
| --- | --- | --- | --- | --- |
| Dependent Variable: Intrusion Distress  Step 1: *R_adj_^2^ = -0.026, p* = 0.630, *df1* = 1, *df2* = 29 | | | | |
| Intercept | 24.28 | 3.38 |  |  |
| IC | 1.67 | 3.43 | 0.091 | 0.630 |
| Step 2: Δ*R^2^ = 0.264, p* = 0.033, *df1* = 3, *df2* =27 | | | | |
| Intercept | 24.28 | 3.00 |  |  |
| IC | 2.34 | 3.07 | 0.126 | 0.452 |
| HR murder | -11.76 | 4.10 | -0.633 | 0.008 |
| HR assault | 11.58 | 4.08 | 0.624 | 0.008 |
| Step 3: Δ*R^2^ =* 0.087*, p* = 0.038, *df1* = 5, *df2* = 25 | | | | |
| Intercept | 24.280 | 2.92 |  |  |
| IC | 1.093 | 3.07 | -0.056 | 0.725 |
| HR murder | -9.946 | 4.16 | -0.536 | 0.025 |
| HR assault | 8.895 | 4.30 | 0.479 | 0.049 |
| Pt emo murder | 0.725 | 4.14 | -0.039 | 0.862 |
| Pt emo assault | 5.443 | 4.14 | 0.293 | 0.200 |

*Note.* All predictor variables are z-standardized.
IC = Inhibitory control, measured at baseline with the Stroop task
Pt emo = peritraumatic emotion rating of respective trauma-analogue movie scenes
HR = heart rate
B = unstandardized estimate, β = standardized estimate, p-value was set to alpha 0.05

**Table S4** *Multiple Hierarchical Regression results for Impact of Event Scores*

|  | B | SE B | *β* | *p* |
| --- | --- | --- | --- | --- |
| Dependent Variable: IES scores Step 1: *R_adj_^2^ = 0.084, p* = 0.062, *df1* = 1, *df2* = 29 | | | | |
| Intercept | 13.02 | 1.98 |  |  |
| IC | 3.92 | 2.02 | 0.340 | 0.062 |
| Step 2: Δ*R^2^ = 0.145, p* = 0.040, *df1* = 3, *df2* =27 | | | | |
| Intercept | 13.02 | 1.88 |  |  |
| IC | 3.76 | 1.92 | 0.326 | 0.061 |
| HR murder | -1.38 | 2.57 | -0.120 | 0.596 |
| HR assault | 5.19 | 2.56 | 0.450 | 0.052 |
| Step 3: Δ*R^2^ =* 0.118 *, p* = 0.148, *df1* = 5, *df2* = 25 | | | | |
| Intercept | 13.024 | 1.95 |  |  |
| IC | 3.603 | 2.04 | 0.312 | 0.090 |
| HR murder | -1.265 | 2.77 | -0.110 | 0.652 |
| HR assault | 4.997 | 2.86 | 0.433 | 0.093 |
| Pt emo murder | -0.591 | 2.76 | -0.051 | 0.832 |
| Pt emo assault | 1.124 | 2.76 | 0.097 | 0.687 |

*Note.* All predictor variables are z-standardized.
IC = Inhibitory control, measured at baseline with the Stroop task
Pt emo = peritraumatic emotion rating of respective trauma-analogue movie scenes
HR = heart rate
B = unstandardized estimate, β = standardized estimate, p-value was set to alpha 0.05

**Table S4a** *Multiple Regression results for Impact of Event Hyperarousal subscale*

|  | B | SE B | *β* | *p* |
| --- | --- | --- | --- | --- |
| *R_adj_^2^ =* 0.140*, p* = 0.046, *df1* = 2, *df2* = 28 | | | | |
| Intercept | 0.3065 | 0.0606 |  |  |
| IC | 0.0298 | 0.0617 | 0.0819 | 0.633 |
| HR assault | 0.1570 | 0.0617 | 0.4315 | 0.017 |

*Note.* All predictor variables are z-standardized.
IC = Inhibitory control, measured at baseline with the Stroop task
HR = heart rate, B = unstandardized estimate, β = standardized estimate, p-value was set to alpha 0.05

**Table S4b** *Multiple Regression results for Impact of Event Intrusion subscale*

|  | B | SE B | *β* | *p* |
| --- | --- | --- | --- | --- |
| *R_adj_^2^ =* 0.0626*, p* = 0.154, *df1* = 2, *df2* = 28 | | | | |
| Intercept | 0.713 | 0.106 |  |  |
| IC | 0.119 | 0.108 | 0.195 | 0.279 |
| HR assault | 0.173 | 0.108 | 0.283 | 0.121 |

*Note.* All predictor variables are z-standardized.
IC = Inhibitory control, measured at baseline with the Stroop task
HR = heart rate, B = unstandardized estimate, β = standardized estimate, p-value was set to alpha 0.05

**Table S4c** *Multiple Regression results for Impact of Event Avoidance subscale*

|  | B | SE B | *β* | *p* |
| --- | --- | --- | --- | --- |
| *R_adj_^2^ =* 0.261*, p* = 0.006, *df1* = 2, *df2* = 28 | | | | |
| Intercept | 0.727 | 0.119 |  |  |
| IC | 0.321 | 0.121 | 0.418 | 0.013 |
| HR assault | 0.264 | 0.121 | 0.343 | 0.038 |

*Note.* All predictor variables are z-standardized.
IC = Inhibitory control, measured at baseline with the Stroop task
HR = heart rate, B = unstandardized estimate, β = standardized estimate, p-value was set to alpha 0.05

**Table S5** *Multiple Hierarchical Regression results for median response time for negative trauma reminders*

|  | B | SE B | *β* | *p* |
| --- | --- | --- | --- | --- |
| Dependent Variable: RTs for negative reminders Step 1: *R_adj_^2^ = 0.182, p* = 0.017, *df1* = 1, *df2* = 29 | | | | |
| Intercept | 585.6 | 15.4 |  |  |
| IC | 39.9 | 15.7 | 0.427 | 0.017 |
| Step 2: Δ*R^2^ = 0.175, p* = 0.007, *df1* = 3, *df2* =27 | | | | |
| Intercept | 585.6 | 14.2 |  |  |
| IC | 39.3 | 14.5 | 0.421 | 0.012 |
| HR murder | -12.0 | 19.4 | -0.225 | 0.288 |
| HR assault | 48.7 | 19.3 | 0.532 | 0.016 |
| Step 3: Δ*R^2^ =* 0.009*, p* = 0.034, *df1* = 5, *df2* = 25 | | | | |
| Intercept | 585.65 | 14.6 |  |  |
| IC | 41.37 | 15.4 | 0.443 | 0.012 |
| HR murder | -12.45 | 20.8 | -0.251 | 0.271 |
| HR assault | 53.44 | 21.5 | 0.572 | 0.020 |
| Pt emo murder | 2.06 | 20.7 | 0.022 | 0.922 |
| Pt emo assault | -11.06 | 20.7 | -0.118 | 0.597 |

*Note.* All predictor variables are z-standardized.
IC = Inhibitory control, measured at baseline with the Stroop task
Pt emo = peritraumatic emotion rating of respective trauma-analogue movie scenes
HR = heart rate
B = unstandardized estimate, β = standardized estimate, p-value was set to alpha 0.05

**Table S6** *Overview of complementary analyses using change scores for all multiple regression models*

| Criterion   Predictors | Intrusion Frequency | Intrusion Vividness | Intrusion Distress | IES Score | IES hyper-arousal | IES intrusion | IES avoidance | RTs negative reminders |
| --- | --- | --- | --- | --- | --- | --- | --- | --- |
| Inhibitory Control | -0.001 | -0.003 | 0.045 | 0.312 | 0.083 | 0.189 | 0.423* | 0.41* |
| *d’* assault | 0.749** | 0.420 | 0.478* | 0.388 | 0.383* | 0.36* | 0.234 | 0.572* |
| *d‘* murder | -0.228 | -.0.21 | -0.28 | -0.072 | - | - | - | -0.002 |
| emotions assault | -0.045 | 0.35 | 0.27 | 0.136 | - | - | - | -0.046 |
| emotions murder | -0.011 | -0.15 | 0.067 | -0.095 | - | - | - | -0.055 |
| Full model | R^2^ = 0.33  *p* = .062 | R^2^ = 0.21  *p* = .283 | R^2^ = 0.32  *p* = .074 | R^2^ = 0.25  *p =* .191 | R^2^ = 0.16  *p =* .091 | R^2^ = 0.17  *p =* .069 | R^2^ = 0.25  *p =* .019 | R^2^ = 0.28  *p =* .021 |

*Note.* Standardized coefficients (beta) are displayed for the full model (step 3 in the hierarchical regression, see results for more details and supplementary material tables S1-5 for all coefficients)
Higher Inhibitory control scores represent lower inhibitory control
**p* < .05 ** *p < .*01
*d’* = change score (heart rate during trauma scene – heart rate during control scene)

**Table S7** *Descriptive Statistics for Change Scores by Gender*

| Gender | N | Mean Murder | Mean Assault | SD Murder | SD Assault |
| --- | --- | --- | --- | --- | --- |
| Female | 23 | 0.150 | 6.65 | 4.26 | 10.4 |
| Male | 8 | -4.27 | -1.75 | 4.37 | 3.58 |

*Note.* Gender: 1 = Female, 2 = Male; SD = Standard deviation; Change score was calculated by subtracting the mean heart rate in beats per minute (BPM) from the control scene from the trauma-analogue scene

**Table S8** *Overview of comparision of HRV measures between movie scenes*

| HRV outcome | df | F | *p* |
| --- | --- | --- | --- |
| RMSSD* | 2, 56 | 0.163 | .850 |
| SDNN | 2, 56 | 0.900 | .412 |
| PNN50 | 2, 56 | 0.453 | .638 |
| LF | 2, 56 | 2.16 | .124 |
| HF | 2, 56 | 0.530 | .592 |
| LF/HF | 2, 56 | 1.74 | .185 |

*Note.* HRV = Heart Rate Variability; RMSSD = Root Mean Square of Successive Differences; SDNN = Standard Deviation of NN Intervals; PNN50 = Percentage of NN Intervals Greater than 50 ms; LF = Low-Frequency Power; HF = High-Frequency Power; LF/HF = Ratio of Low-Frequency to High-Frequency Power.
df = degrees of freedom, F = F-statistic, p-value at .05; * for RMSSD the 2 min sections of the movie were used, whereas for all other analyses we used the original movie scene length of 9min

**Table S9** *Descriptive statistics of HRV variables by movie scene*

| Scene | RMSSD* |  | SDNN | PNN50 | LF | HF | LF/HF |
| --- | --- | --- | --- | --- | --- | --- | --- |
| **Murder** | 39.3 (13.1) |  | 55.2 (16.6) | 18.9 (13.0) | 1.13 (0.423) | 0.761 (0.342) | 1.80 (1.05) |
| **Control** | 38.7 (12.2) |  | 54.9 (16.7) | 17.5 (11.6) | 1.16 (0.446) | 0.766 (0.397) | 1.94 (1.20) |
| **Assault** | 39.4 (13.2) |  | 59.1 (22.2) | 18.3 (12.1) | 1.01 (0.541) | 0.708 (0.324) | 1.63 (1.09) |

*Note.* HRV = Heart Rate Variability; RMSSD = Root Mean Square of Successive Differences; SDNN = Standard Deviation of NN Intervals; PNN50 = Percentage of NN Intervals Greater than 50 ms; LF = Low-Frequency Power; HF = High-Frequency Power; LF/HF = Ratio of Low-Frequency to High-Frequency Power.
* for RMSSD the 2 min sections of the movie were used, whereas for all other analyses we used the original movie scene length of 9min, Standard Deviations displayed in brackets

**References for Supplementary Material**

1. Herz, N., Bar-Haim, Y., Holmes, E. A. & Censor, N. Intrusive memories: A mechanistic signature for emotional memory persistence. *Behav. Res. Ther.* **135**, 103752 (2020).

2. Spitzer, R. L., Kroenke, K., Williams, J. B. W. & Group, and the P. H. Q. P. C. S. Validation and Utility of a Self-report Version of PRIME-MD: The PHQ Primary Care Study. *JAMA* **282**, 1737–1744 (1999).

3. Spielberger *et al.* Manual for the State-Trait Anxiety Inventory. *Palo Alto, CA: Consulting Psychologists Press.* (1983).

4. James, E. L. *et al.* The trauma film paradigm as an experimental psychopathology model of psychological trauma: intrusive memories and beyond. *Clin. Psychol. Rev.* **47**, 106–142 (2016).

5. *Psychology Software Tools Inc., Pittsburgh, PA, USA).* (2016).

6. *BIOPAC Systems Inc.,MP150 Data Acquisition System., Goleta, CA, USA*. (2023).

7. Noé, G. *Irréversible, Nord-Ouest Films*. (2002).

8. Jarmusch. *Paterson, Amazon Studios*. (2016).

9. Marchewka, A., Żurawski, Ł., Jednoróg, K. & Grabowska, A. The Nencki Affective Picture System (NAPS): Introduction to a novel, standardized, wide-range, high-quality, realistic picture database. *Behav. Res. Methods* **46**, 596–610 (2014).

10. Menon, V., Adleman, N. E., White, C. D., Glover, G. H. & Reiss, A. L. Error‐related brain activation during a Go/NoGo response inhibition task. *Hum. Brain Mapp.* **12**, 131–143 (2001).

11. Scarpina, F. & Tagini, S. The Stroop Color and Word Test. *Front. Psychol.* **8**, 557 (2017).

12. Weiss, D. S. Cross-Cultural Assessment of Psychological Trauma and PTSD. *Int. Cult. Psychol. Ser.* 219–238 (2007) doi:10.1007/978-0-387-70990-1_10.

13. Vollmer, M. HRVTool – an Open-Source Matlab Toolbox for Analyzing Heart Rate Variability. *2019 Comput. Cardiol. (CinC)* Page 1-Page 4 (2019) doi:10.22489/cinc.2019.032.

14. Blanchard, E. B., Hickling, E. J., Galovski, T. & Veazey, C. Emergency room vital signs and PTSD in a treatment seeking sample of motor vehicle accident survivors. *J. Trauma. Stress* **15**, 199–204 (2002).

15. Chou, C.-Y., Marca, R. L., Steptoe, A. & Brewin, C. R. Heart rate, startle response, and intrusive trauma memories. *Psychophysiology* **51**, 236–246 (2014).

16. Bryant, R. A., Creamer, M., O’Donnell, M., Silove, D. & McFarlane, A. C. A Multisite Study of Initial Respiration Rate and Heart Rate as Predictors of Posttraumatic Stress Disorder. *J. Clin. Psychiatry* **69**, 1694–1701 (2008).

17. Beutler, S. *et al.* Trauma-related dissociation and the autonomic nervous system: a systematic literature review of psychophysiological correlates of dissociative experiencing in PTSD patients. *Eur. J. Psychotraumatology* **13**, 2132599 (2022).

18. Melo, H. M. *et al.* Ultra-short heart rate variability recording reliability: The effect of controlled paced breathing. *Ann. Noninvasive Electrocardiol.* **23**, e12565 (2018).

19. Ruangsuphaphichat, A. *et al.* Test-retest reliability of short- and long-term heart rate variability in individuals with spinal cord injury. *Spinal Cord* **61**, 658–666 (2023).

20. Apšvalka, D., Ferreira, C. S., Schmitz, T. W., Rowe, J. B. & Anderson, M. C. Dynamic targeting enables domain-general inhibitory control over action and thought by the prefrontal cortex. *Nat Commun* **13**, 274 (2022).

21. Depue, B. E., Orr, J. M., Smolker, H. R., Naaz, F. & Banich, M. T. The Organization of Right Prefrontal Networks Reveals Common Mechanisms of Inhibitory Regulation Across Cognitive, Emotional, and Motor Processes. *Cereb Cortex* **26**, 1634–1646 (2016).

22. Wessel, J. R. & Anderson, M. C. Neural mechanisms of domain-general inhibitory control. *Trends Cogn. Sci.* **28**, 124–143 (2024).

23. Hammar, Å., Schmid, M. T., Petersdotter, L., Ousdal, O. T. & Milde, A. M. Inhibitory control as possible risk and/or resilience factor for the development of trauma related symptoms–a study of the Utøya terror attack survivors. *Appl. Neuropsychol.: Adult*, 1–13 (2023).

24. Joyal, M. *et al.* Characterizing emotional Stroop interference in posttraumatic stress disorder, major depression and anxiety disorders: A systematic review and meta-analysis. *Plos One* **14**, e0214998 (2019).
